# Ethical frameworks for obtaining informed consent in tumour profiling: an evidence-based case for Singapore

**DOI:** 10.1186/s40246-017-0127-1

**Published:** 2017-12-08

**Authors:** Yasmin Bylstra, Tamra Lysaght, Jyothi Thrivikraman, Sangeetha Watson, Patrick Tan

**Affiliations:** 1 POLARIS, Genome Institute of Singapore, Agency for Science, Technology and Research, Singapore, Singapore; 2 SingHealth Duke-NUS Institute of Precision Medicine, Singapore, Singapore; 3 Inherited Cardiac Clinic, National University Hospital, Singapore, Singapore; 4 Centre for Biomedical Ethics, Yong Loo Lin School of Medicine, National University of Singapore, Singapore, Singapore; 5 Cancer and Stem Biology, Duke-NUS Medical School, Singapore, Singapore; 6 Biomedical Research Council, Agency for Science, Technology and Research, Singapore, Singapore

**Keywords:** Tumour profiling, Informed consent, Genomic data sharing, Germline mutations

## Abstract

**Background:**

Genomic profiling of malignant tumours has assisted clinicians in providing targeted therapies for many serious cancer-related illnesses. Although the characterisation of somatic mutations is the primary aim of tumour profiling for treatment, germline mutations may also be detected given the heterogenous origin of mutations observed in tumours. Guidance documents address the return of germline findings that have health implications for patients and their genetic relations. However, the implications of discovering a potential but unconfirmed germline finding from tumour profiling are yet to be fully explored. Moreover, as tumour profiling is increasingly applied in oncology, robust ethical frameworks are required to encourage large-scale data sharing and data aggregation linking molecular data to clinical outcomes, to further understand the role of genetics in oncogenesis and to develop improved cancer therapies.

**Results:**

This paper reports on the results of empirical research that is broadly aimed at developing an ethical framework for obtaining informed consent to return results from tumour profiling tests and to share the biomolecular data sourced from tumour tissues of cancer patients. Specifically, qualitative data were gathered from 36 semi-structured interviews with cancer patients and oncology clinicians at a cancer treatment centre in Singapore. The interview data indicated that patients had a limited comprehension of cancer genetics and implications of tumour testing. Furthermore, oncology clinicians stated that they lacked the time to provide in depth explanations of the tumour profile tests. However, it was accepted from both patients and oncologist that the return potential germline variants and the sharing of de-identified tumour profiling data nationally and internationally should be discussed and provided as an option during the consent process.

**Conclusions:**

Findings provide support for the return of tumour profiling results provided that they are accompanied with an adequate explanation from qualified personnel. They also support the use of broad consent regiments within an ethical framework that promotes trust and benefit sharing with stakeholders and provides accountability and transparency in the storage and sharing of biomolecular data for research.

**Electronic supplementary material:**

The online version of this article (10.1186/s40246-017-0127-1) contains supplementary material, which is available to authorized users.

## Background

Advances in genomic technologies and declining costs of sequencing have expanded opportunities to conduct genetic profiling of diseased cells routinely. In oncology, molecular testing of tumours, such as breast cancer, has been practiced for over 20 years. These tests can define the tumour subtype, which has important implications for the selection of therapeutic options. However, testing has since evolved to differentiate both heritable (germline) and tumour-specific (somatic) mutations in tumours. These developments have revolutionised cancer care and bridged a new era of chemotherapy and targeted treatments.

The value in delineating somatic and germline genomics for therapeutic purposes has already been demonstrated with the efficacy of ADP-ribose polymerase (PARP) inhibitors for patients with a germline BRCA mutation [1]. In parallel, next generation sequencing (NGS) platforms are also integral to translational cancer research in identifying and validating promising new biomarkers for the development of cancer treatment. Worldwide collaborative efforts, such as The Cancer Genome Atlas (TCGA) and the International Cancer Genome Consortium (ICGC), have catalogued the genomic landscapes of thousands of tumours. In such settings, germline DNA has been routinely collected for comparative analysis with tumour DNA from the same patient to distinguish unambiguously true somatic mutations from rare germline polymorphisms. However, clinical practice is currently shifting towards a preference of routinely sequencing a patient’s tumour tissue alone, to characterise its molecular profile: reasons for this preference include cost reduction and simplifying the logistics of sample collection [2, 3]. Sequencing a patient’s tumour tissue alone, in the absence of a matched germline sample, challenges accurate delineation of somatic versus germline mutations due to the heterogeneous nature of mutations observed in tumours.

Until recently, there was no clear guidance on whether or how findings that only imply germline variations from genomic tumour profiling should be returned to patients. International governance bodies, such as the World Health Organization (WHO) and the Organisation for Economic Co-Operation Development (OECD), as well as national regulatory authorities around the world, have issued guidelines on the management of genetic databases; although few have developed explicit guidance on the return of incidental findings [4]. These guidance documents address the return of germline findings known to have health implications for individual participants as well as their genetic relations. However, none address the situation where tumour profiles results in a potential, but unconfirmed germline finding. In response to the increasing utility of tumour-only testing in clinical practice, the American Society of Clinical Oncology (ASCO) updated their policy statement to include recommendations that the patients should be made aware of the possible detection of germline mutations [5]. However, the implications of these recommendations in day-to-day clinical practice have not yet been fully explored, particularly in Asia.

In addition to the uncertainty around managing incidental findings, the need for sharing biomolecular data internationally is increasingly being recognised as critical to understanding the role of genetics in oncogenesis and delivering more effective target therapies for cancer [6]. However, researchers require common guidelines to ensure accountability and ethical oversight for the protection of patient data that is shared between institutions and across international borders [6, 7]. In 2014, the Global Alliance for Genomics and Health (GA4GH) published the *Framework for Responsible Sharing of Genomic and Health-Related Data* that establishes a set of foundational principles for sharing genomic and health-related data [8]. According to this framework, best practices for sharing genomic and health-related data should ‘promote and protect respect for the commitment to informed consent’ as the foundational principle that underlies the ethical conduct of all research involving human subjects [9]. However, while intended to facilitate compliance with international norms, this framework should also be interpreted in a manner that recognises local cultural practices and the different contexts for storing and sharing data.

In developing an ethical framework that is culturally appropriate and sensitive to local norms, systems and preferences, we initiated a qualitative study in Singapore to explore the understandings, attitudes and preferences that cancer patients and clinicians have towards the return of results of tumour profiling tests as well as the usage and sharing of the data for research purposes. Singapore is an ethnically diverse and multi-cultured country of 5 million situated in South East Asia. As most published studies on patient understandings of genetic testing have focused predominantly on Caucasian populations, there is value in gaining further insights from a diverse Asian perspective. Currently, there are no laws in Singapore to protect patients against employment and insurance discrimination due to their genetic status. Any framework for obtaining informed consent in this context should also take into account local concerns about genetic discrimination and trust in governance mechanisms that oversee the storage and sharing of genome data.

## Methods

The study was designed with the aims of exploring and describing the attitudes, understandings and preferences that clinicians and cancer patients have towards participation in tumour profiling research, storage and sharing of tumour genetic data, and the return of tumour profiling results. To achieve these aims, the study design employed qualitative research methods, which are useful for documenting and explaining variation in a wide range of views, needs, values, practices and beliefs [10]. These methods are not designed to estimate proportions in a wider population, quantify relationships between pre-determined variables or provide a single representative or average view or opinion [11]. However, they are particularly useful for policy development and for the design and delivery of health care and are especially well-suited to exploring the understandings and attitudes towards highly complex concepts and subjects that cannot be fully captured with quantitative methodologies.

Evidence for this study was collected from semi-structured qualitative interviews with patients and clinicians at the National Cancer Centre Singapore (NCCS). This method was chosen to provide a contextualised dataset that focused on the specified issues and could generate themes from clearly defined, homogeneous populations within an already known context [10, 12]. Ethics approval for this protocol was obtained from the Domain Specific Research Board (DSRB) of SingHealth on 9 July 2015 to conduct up to 40 semi-structured interviews with SingHealth staff and cancer patients: (2015/2522).

### Recruitment

Participants were selected using purposive sampling techniques to allow for greater flexibility in targeting informants and capture a broad range of perspectives [13] until thematic saturation was reached (i.e. no new themes were emerging from the data analysis to justify continued recruitment) [14]. To identify relevant clinicians, clinical members of the research team provided a list of 25 key oncologists at the NCCS. Three emails were sent to these individuals over 3 weeks starting at the end of July 2015. From these emails, six clinicians agreed to be interviewed, resulting in a response rate of 24%. To increase the sample size, a second email invitation was sent to an additional 59 SingHealth oncology staff in early August. Of these, 15 responded and five agreed to participate. This process resulted in a total of 11 participants being recruited from a pool of 74 contacts (a response rate of 14.8%). No further attempts were made to increase the sample size after the data were thematically saturated at 11 interviews. These interviews were conducted between July and September 2015 on the phone and face-to-face based on the preferences of participants.

For the patient group, eligible participants were recruited from the waiting room at the NCCS with the assistance of staff at the registration desk in the public clinic as well as the nurses in the private clinic. Participants were offered a $50 supermarket voucher as fair compensation for their time. Of the 28 patients approached, only three declined to be interviewed resulting in a sample of 25 informants. The much higher response rate of 89.3% in this group was likely due to the support of their treating physician, being present onsite, face-to-face recruitment and compensation. The patient interviews were conducted in October 2015.

### Interview protocol

An interview protocol for both groups was developed from the issues identified in a literature review (see Additional file 1). The clinician interviews were structured around three key issues to describe their attitudes and preferences towards (1) how information on the cancer diagnosis and the role of genetics (if any) is delivered to the patient; (2) delivery of the tumour profiling test results; and (3) the type of informed consent document needed to explain storing, sharing and withdrawal of tumour profile data. The interview protocol for the patient group was designed to explore participant attitudes, understandings and preferences of key issues including the purpose of the test; preferred linguistic labels and options of delivering the informed consent; the test procedures (sharing, storing and re-contact for additional research); perceived benefits and risks; ideas of altruism and solidarity; attitudes towards withdrawal options; and role of family and medical professionals in decision-making. The interview guide was piloted with one patient in the colon cancer clinic before full data collection proceeded.

### Data analysis

All interviews were digitally recorded, transcribed verbatim and analysed using qualitative content analysis to identify, categorise and interpret key themes in relation to the consent process for storing and sharing of biomolecular data. Transcripts were read multiple times by the interviewer along with two study team members (YB and TL) to identify major themes and sub-themes. These themes and sub-themes were discussed together by the three study team members to corroborate categories and placement of relevant quotes. A coding frame was developed using these themes, which a fourth research applied to the data using NVivo© software (QSR International). Reliability was checked with two of the team members (YB and TL) independently coding pages of randomly selected interview transcripts. Agreement was measured using Cohen’s kappa coefficient [15]. Demographics (age, ethnicity, gender and type of cancer diagnosis) were also collected for each patient and analysed using summative descriptive statistics (averages, median and frequencies).

## Results

From August to October 2015, 11 clinicians (7 oncologists, 2 cancer genetic specialists and 3 palliative care doctors) were interviewed at the NCCS. Patients in both the private and public breast cancer clinics were recruited. In total, 25 patients were interviewed (see Table 1 for demographic descriptions of patients).

**Table 1:** Demographics of breast cancer patients interviewed

Patient ID	Age	Ethnicity	No. of patients
1	43	Bangladeshi	2 (8%)
2	59	Bangladeshi	
3	39	Chinese	10 (40%)
4	46	Chinese	
5	49	Chinese	
6	52	Chinese	
7	52	Chinese	
8	54	Chinese	
9	55	Chinese	
10	59	Chinese	
11	60	Chinese	
12	60	Chinese	
13	58	Filipino	1 (4%)
14	40	Indian	6 (24%)
15	40	Indian	
16	53	Indian	
17	58	Indian	
18	64	Indian	
19	69	Indian	
20	35	Malay	3 (12%)
21	63	Malay	
22	68	Malay	
23	27	Malay-Chinese	1 (4%)
24	50	Pakistani	1 (4%)
25	46	Vietnamese	1 (4%)
Total no. of patients			25

The average age of those interviewed was 52, with a median age of 54 years (ranging from 27 to 69). The majority were ethnic Chinese (10), with Indian (6) being the next largest group. The process of obtaining informed consent for the tumour profiling tests was discussed to explore patient understandings of consent and their attitudes towards the return of results and sharing of biomolecular data for research. From these discussions, broad themes emerged that described several issues concerning participant understandings of cancer genetics and tumour testing results, therapeutic misconceptions, privacy and confidentiality. Results were supportive of a broad consent model in these contexts.

### Current practice for discussing tumour profiling tests

To frame the clinical context of discussions around tumour profiling tests, the communication of this information was explored with both clinicians and patients. Clinicians indicated that their conversations about tumour testing with their patients were heavily simplified and that cancer genetics terms, such as somatic and germline mutations, were not distinguished. Instead, information regarding the test was briefly summarised by explaining that results would clarify treatment options. Clinicians explained that family history was raised if appropriate, or if patients had any concerns, referrals would be made to the cancer genetic clinics for further review.

‘And if you tell them that they have this mutation, it means you can receive this drug. It will work on you or not work on you. I think about, that is how we explain it.’ (Clinician 1)

Likewise, patients also indicated that discussions around genetics were limited, although some recalled being asked about their family history of cancer. When explored further about the information needs the patients desired, the response ranged widely. Some preferred brief information whereas others wanted to be well-informed. In discussions with patients about cancer genetics and tumour profiling, it became apparent that they had very limited understandings of these concepts and many appeared confused about somatic and germline genetics when raised. Cancer was generally perceived as being primarily hereditary, even by one highly educated patient who had a background in health communications:

‘Generally as a lay person we are more inclined to look at family genetics. We would not think about, what you told me. When I say mutations, I was referring to the family genes you mention to me…I know every cancer cell is a mutation… normally when we talk about mutations, we talk about family history.’ (Patient 9)

Clinicians interviewed also agreed that patients had limited understandings of genetics, mutations and cancer development. Some suggested that these limitations would even apply to clinicians who did not specialise in cancer genetics:

‘It is does not stop at patients. Even physicians. I’ve had so many…mis…even from physicians referring to me you can understand that their grasp of it is very low between driver and passenger mutations, between actionable and not actionable. So you can’t blame patients for not knowing this.’ (Clinician 11)

It emerged from both clinicians and patients that the concepts around tumour profiling were complex and would require in depth explanations for an appropriate level of comprehension to be achieved.

### Return of tumour profiling results

Current practices were explored regarding the return tumour test results. Some clinicians gave their patients the option of receiving the results as standard practice and others believed that the results should be returned to both the participant and their treating oncologist without any opportunities for either party to opt out. However, not all clinicians agreed with this approach, as with many clinical tests, there can be uncertainty around the results and their implications for treatment. For example, the pathogenicity of a variant may be unclear (known as ‘variant of unknown significance’) or a variant may be reported for research opportunities, such as clinical trials, rather than for immediate clinical benefit.

‘…if you just give consent and say, “Here are the clinical one and these are research ones”, and you just say that, nothing else, no support about possible uncertainty. And the patient comes back and says “What does this mean?”, and the doctor says this means “uncertainty”. And the patient says “Oh, this is something you never told me, we [didn’t] know and I don’t really want this.’ (Clinician 2)

As the tumour test panels can contain genes that are associated with hereditary conditions, there is the possibility that mutations in these genes derive from germline origin and have familial implications. The prospect of identifying germline incidental findings generated conflicting ideas amongst clinicians. Some clinicians discussed the possibility of discrimination that could follow from germline analyses and suggested that more legal protections, such as the Genetic Information Nondisclosure Act (2008) in the USA, should be in place before these types of tests are introduced widely into precision oncology. Hence, there were stated preferences for tumour profiling tests to exclude analysis of genes that could imply germline mutations until measures were in place. Others felt that patients should know, but should be referred to another healthcare professional trained in discussing germline implications:

‘I don’t think I can hide this from the patient. You have to tell the patient. Patient’s interest for me to tell them to get tested. This has implications for you. I will send you to a trained cancer genetics oncologist or counsellor or whatever to sort this out. It should not be done in my clinic. It has to be someone trained.’ (Clinician 10)

Most of the clinicians interviewed were clear about their professional boundaries and limitations. They understood that further evidence would be required to support the origin of a germline finding and that this was usually beyond their role as an oncologist. They also agreed that they did not have sufficient training to counsel and educate patients about germline implications.

Patients, on the other hand, were clearer in their preferences to receive their tumour profiling results. They were informed that this information could include a risk to hereditary conditions, such as breast cancer or cardiac conditions, or new treatments currently under research. Yet they were clear that they wanted to know about these findings even if they had implications outside their own cancer diagnosis. They indicated that they were open to receiving any information that may help them understand why they were diagnosed with cancer or advance their treatment:

‘I'm completely okay with that. So if there is a way that we can receive then why not. We would like to have the information.’ (Patient 21)

Many patients interviewed indicated a strong preference to receive results with the provision that their oncologist or another trusted healthcare professional explain the implications of the results. They agreed that receiving such information independent of any explanation could create unnecessary confusion or worry. Therefore, if the option of returning tumour tests is available to patients, the inclusion and management of such incidental findings must also be considered.

### Data sharing

Significant to the advancements in research for new therapies is the generation and accessibility of large and diverse genomic data sets. The possibility of sharing the genomic data obtained from the tumour tests with external researchers, both locally and internationally, was discussed with clinicians and patients. Both groups expressed the view that personal information had to be delinked from the stored data before being shared with third parties. Some clinicians also felt that the socio-political culture in Singapore would mean that few patients would be overly concerned about the storage and sharing of their results, and that they would likely consent on the basis of assurances that their personal data would be kept confidential in compliance with local laws and regulations:

‘We in Singapore are not so worried about big brother or privacy like you in the West. We are used to big brother. We are a democracy, but have an overlay of authoritarianism…we are used to having government know about us and having access to our information. Singaporeans will not be bothered about the storing and sharing of data.’ (Clinician 6)

These cultural views were shared amongst some of the patients interviewed who felt that there were sufficient safeguards in Singapore to protect privacy and prevent the data from being disclosed inappropriately and misused, such as the Personal Data Protection Act (2012). However, some patients were concerned about possible misuses, particularly with respect to the potential for discrimination:

‘If it is only for the purpose of research it is okay. There is no problem. But if for whatever reason my name will be shown and published in a publically available media, okay then basically I need to see it and basically I need to have a positive consent for myself before it is published.’ (Patient 6)

Some of the clinicians interviewed expressed concerns about sharing patient data with research institutions in other countries that lacked the accountability of publicly funded institutions like in Singapore. It was also expressed that sharing participant data for profit-orientated research might create mistrust between oncologists and their patients if such information were revealed at a later point. Such perceptions of ‘profit-orientated’ institutions being driven towards the commercial development of products for private benefit conflicted with views of genome research as a ‘public good’.

On the other hand, the patients interviewed did not draw strong distinctions between public and private goods, although the possibility of commercial products being developed from shared tumour profile data was not raised with these informants either. All patients interviewed appeared to understand that they would not benefit directly from the sharing of tumour profile data. However, their willingness to allow their data to be stored and shared with other institutions was sometimes premised on an understanding that the research was aimed at benefiting future cancer patients:

‘I feel very excited that someone can look at this and figure it out. And if they can figure it out here or in Argentina, I don’t really care. It is going to be helpful to other people with a similar tumor profile I would actually be very interested in donating my tumor to science.’ (Patient 14)

There were also indications amongst clinicians that patients would agree to participate in research altruistically if they believe it had the potential to benefit patients in future as a societal good:

‘I think a lot of patients will do it for altruistic reasons.. most patients will do it. But I think they don’t want to be made to feel as if they are guinea pigs. And so I think that’s the balance you want to strike. I think you might want to say “Look whatever profits we get from these cell lines, we will donate it back to cancer”. They don’t feel as if they are being taken for granted and they are helping the future.’ (Clinician 9)

The reciprocation of indirect benefits back to the cancer community would likely be a strong moral justification for sharing tumour profile data. Most patients interviewed in this study did not have a family history, many wanted to understand why they had developed cancer, and were trying to make sense of their diagnosis. The possibility of having those questions answered through increased knowledge of the causes and pathology of cancer was highly valued, and patients could view themselves as contributing to that cause as a benefit. Trust in the potential to generate further knowledge around cancer causation may provide the strongest moral justification for consenting to sharing data under a broad consent regiment.

### Broad consent as a model

Ensuring that patients understand test outcomes and documenting preferences in both research and clinical settings is conditional for informed consent. The process of obtaining informed consent from patients to take part in the tumour profiling tests was discussed with patients and clinicians. These discussions centred on the length and complexity of the consent form, the management of test results and incidental findings, participation in research and sharing of biomolecular data, the type of preferred consent and withdrawal options. In general, both patients and clinicians preferred that information be provided within a broad or blanket consent regiment with an option to withdraw from the research.

Clinicians were generally familiar with the various types of consent regiments (Table 2), and all but one clinician felt that a blanket or broad consent would be most appropriate in this setting. With respect the usage of tumour profiling data for research, one clinician preferred a categorical consent out of concerns for sharing genetic data outside cancer-related research. However, most of the clinicians felt that documentation with multiple consent tiers would be confusing to patients and cumbersome to manage:

“It would be more easier for scientists or researchers to get the one that the patient already say “Okay, I agree you can use it freely for research” and cover all the parameters with the patient. It is easier for the researcher. For the patient, they will feel “Why I have to consent for so many things?” (Clinician 2)

Patients on the other hand were, at times, confused about the concept of informed consent and the different types of consent needed careful explaining. As discussed above, although most patients preferred to receive any potential incidental findings, this preference still needs to be documented during the consent process.

**Table 2:** Glossary of terms for informed consent

Types of consent	
Implied consent	Whereby consent is not explicitly sought from participants to use their samples in research.
Blanket consent	Consent that is sought from the participant once, either at or prior to sample collection, for use in any and all future research without the need obtain any further consent.
Broad consent	Consent that is sought from the participant once, either at or prior to sample collection, for use in any and all research without the need obtain further consent from the participant, who then delegates their decision making authority to an IRB (or another institution) for specific research projects.
Categorical consent	Consent that is sought from the participant to use samples in particular categories of research, and may include an option that allows researchers to recontact participants for consent to use samples outside of nominated areas of research.
Specific consent	Consent that is sought from the participant to use samples in specific research projects only, and may include an option that allows researchers to recontact participants for consent to use samples in other projects.
Tiered consent	Provision of multiple options for participants to choose the type of consent they wish to provide.
Types of consent methods	
Opt out	Whereby consent is not explicitly sought for a given action, but participants are informed about the option to withdraw.
Opt in	Whereby verbal or written consent is explicitly sought from the participant to use samples in research.
Types of withdrawal options	
Tiered withdrawal	Whereby participants are given numerous options to withdraw in varying degrees. I.e. to withdraw from further contact while leaving samples and data in the study, or withdraw samples while leaving data, or withdraw all samples, personal information and discontinued use of data
Single withdrawal	Whereby participants are given the option to either continue participation or withdraw completely.

As patients displayed a limited comprehension of tumour testing and the clinicians expressed they had limited time, additional support such as a dedicated coordinator or counsellor to explain the test in detail and facilitate informed consent was suggested:

‘It may be better if someone else that is trained can do it… you just need a trained counsellor or a trained coordinator, research coordinator who is trained to explain it. And then obviously, we have to take the consent, we sort of don’t have to go through the details of explaining. We just wrap up and answer any specific questions or concerns.’ (Clinician 1)

While some clinicians agreed that a facilitator would be helpful, in contrast others felt that the responsibility to explain the results to participants should lie with the oncologist who has professional obligations to stay updated on current evidence of best practice:

‘They can provide additional information to assist the oncologists, but I think it is incumbent on the individual oncologist to know the information. Because at the end of the day they are physicians; it is their responsibility to keep up to date. Genomics is so much a part of oncology that you have to know.’ (Clinician 7)

While patients supported the assistance of a dedicated co-ordinator or counsellor, there were some concerns about a nurse being able to adequately answer questions, given the perceived complexity of content in the consent form:

‘Definitely it's not the nurse; doctor should be fine, if they have the time, looking at the patients. So I think a neutral person before this test carried out. I think it would be much better, so that they can understand. But the words are pretty tough here for people to understand.’ (Patient 16)

There was recognition from patients and clinicians that support from an additional healthcare professional would allow more time for questions, which may not currently be possible in busy clinical settings. The costs of appointing such a professional in a clinical context were not explored with the informants, although the findings suggest that the existing infrastructure would not be well-equipped to absorb those costs.

## Discussion

This paper reports on qualitative research with cancer patients and oncology clinicians in Singapore to explore current practices with tumour profiling tests and consent preferences for the return of results and sharing of their tumour profiling data. The results should be interpreted within the limitations of the study; notably, the small sample size, low response rates from clinicians, and restriction to breast cancer patients interviewed at a single site in Singapore limits the generalisability of results to facilities outside of this context. However, the aim of this study was not to produce generalizable findings about patients and clinicians everywhere, but to explore and describe the perspectives, understandings and attitudes of stakeholders who will likely engage and contribute to the expansion of tumour profiling tests from within the healthcare settings of Singapore. Thus, findings are informative for obtaining consent in these contexts and may be relevant to other types of genome-related research in Singapore and beyond. From the analysis, three major themes emerged: limited comprehension of cancer genetics and the consent process indicating that decision support is required; the consent preferences regarding the return of test results and usage of tumour profiling data for research; and the issues of trust and accountability in relation to research involvement. These findings are discussed in further detail below.

### Decision support for obtaining consent

One significant finding that emerged from the data was the limited comprehension that patients may have of cancer genetics and implications of the tumour profiling results. It was also evident confusion arose around the concept of consent and preferences to obtaining consent. These findings are not limited to Singapore, as previously reported evidence is suggestive of limited comprehension amongst participants in genome research of genomics and cancer genetics [16–19]. In addition, comprehension limits can also be complicated by the multi-lingual context and cultural beliefs within local healthcare settings that may contribute to the varied understandings of genes and inheritance in cancer development [20]. Although cancer healthcare professionals interviewed were aware of the possibility tumour testing panels contained genes associated with hereditary conditions, there was no indication that this was discussed with their patients. In fact, if there were any indications of hereditary implications, clinicians made referrals to inherited cancer services.

Recently, recommendations from both ASCO and National Comprehensive Cancer Network (NCCN) support the view that patients should be informed about the potential of tumour profiling results inferring hereditary conditions, as well as the potential benefits, limitations and risks prior to the test taking place [5, 21]. Clinicians stated that they often lacked the time to explain medical protocols and outcomes in detail with their patients, and indicated that would they would not have time to provide lengthy explanations of the tumour profile tests. This reality of the local healthcare setting suggests that additional resources would be required to support the consent process. In research settings, dedicated co-ordinators are frequently appointed to support the recruitment and consent process. In the context of genomic research, some scholars have recommended the appointment of trained genetic counsellors to deliver the consent documentation and explain to participants the implications of consenting as they are trained to discuss issues related to germline genetic tests [17, 22].

With the significance of tumour profiling panels containing genes associated with hereditary risk, it has been proposed that the role of a genetic counsellor to deliver counselling around such tests should become more predominant in oncology as consultations parallel germline testing [21]. In countries where genetic counselling services are limited, such as Singapore, the involvement of a trained co-ordinator was proposed. With a few exceptions, the clinicians were generally supportive of a dedicated clinical co-ordinator or researcher being available to explain the consent documentation in detail and take the written consent from participants. The additional support to assist with consent will require funding to compensate for the cost of this service. This responsibility may extend to the role of existing hospital employees to assist with the consent or this service could be included in the cost of the tumour profiling test. Institutions will need to consider how the cost of such support can be absorbed.

### Consent preferences for returning results and data sharing

Recommendations provided by the ASCO and NCCN also emphasise that patients should be given the opportunity to opt out of receiving possible incidental germline findings. In addition, for those patients interested learning more about germline origin should be further investigated for their pathogenicity [5, 21]. The obligation to return research results and incidental findings to patients in genetics research is contested and currently lacks consensus [4]. In Singapore, there are currently no laws that create legal duties for clinicians or researchers to return results or incidental findings to participants; nor are there any explicit rights ‘not to know’. As recommended, results from this study also suggest that participants should be given the option to receive the results of their tumour profiling test, and be made aware of the potential for incidental findings during the consent process.

However, discussions became more complex regarding how incidental findings should be highlighted if patients opted to receive a copy of their tumour profiling results. While one clinician interviewed suggested the removal of those genes with germline implications from the tumour test report, most clinicians and patients were generally comfortable with the inclusion of these genes providing that they were accompanied with an adequate explanation. In recommendations for the delivery of tumour profiling results, it has been suggested that oncologists draw on the expertise of genetics specialists to assist with the interpretation and discussions of those findings with participants [21, 23]. The possibility of incidental germline findings and genetic discrimination also emerged from interview data. As there are currently no laws in Singapore to protect patients against employment and insurance discrimination due to their genetic status this approach also justifies a role for genetics specialists having a role to raise awareness of such issues. Therefore, the possibility of incidental findings being revealed with the return of tumour profiling results should be acknowledged along with ensuring that participants are referred to relevant specialists to validate the findings and take action where appropriate.

There are currently no guidelines or recommendations on how these preferences towards the return of results should be captured. It is also apparent from literature that the consent framework and clinical processes to inform patients and document preferences about the hereditary implications of tumour profiling tests remain uncertain. Results from the present study suggest that clinicians and cancer patients would prefer a simple model where consent is given just once. This model was preferred by clinicians because they lacked the time needed to explain adequately the implications of multiple consent options. Some also felt that participants would not fully comprehend different categories of research and would be happy to provide a one-off consent. They also specified that if testing revealed information beyond somatic implications that this should be captured separately, by including a tick box, to consenting for tumour profiling to be performed.

This view also extended to preferences around the usage of tumour profiling data for research applications. Patients were generally unconcerned about the provision of their data providing it was shared with external researchers in a de-identified format, and in compliance with Singapore laws and regulations. Similar to the many other studies published previously, the findings indicated support for a broad consent model that delegates decision-making authority to an independent oversight body, such as an ethics review committee or institutional review board [24, 25].

### Trust and governance

From the results of this study, it is clear that the storage and sharing of tumour profiling data cannot be ethically justified as merely an exercise of personal autonomy when the informed consent of participants is inherently limited. Even with the support of dedicated research staff and a simplified consent process, the degree to which cancer patients can be truly informed of the implications for consenting to the storage and sharing of these data with researchers in Singapore and abroad is uncertain. Thus, it is important to ensure that other measures are in place to protect participants from unnecessary harms and that their data is shared within the morally accepted parameters of the consent. In short, participants must be able to trust that their data will be protected and used for the purposes they consented to.

A lack of trust with clinicians and patients would have significant implications for the value of biomolecular databanks specific to the health needs of the Singaporean population. Storage and sharing these data with external researchers will be key to fostering research and ensuring the widest public health benefits [6]. The potential for these benefits justifies the enormous public resources that are invested in genomic databanks and their purpose as a public good. Maintaining trust in this public good not only requires security measures to protect the data of participants, but will also require transparency in how the data are accessed and how social and economic benefits are distributed [26]. Any intention to privatise these benefits should be disclosed to participants prior to consenting and policies should be in place to restrict access to the data for purposes participants have consented to.

The results of this study support the adoption of a broad consent model where participants would not consent to specific projects or types of research. However, they also suggest that participants would consent altruistically on the condition that their data is used for research that has the potential to benefit other cancer patients in the future. This finding is supported in the literature with other evidence that solidarity with future patients incentivises participation in research that is unlikely to have direct benefits for participants [27]. The solidarity principle forms the basis of ethical arguments that justify the use of broad consent regiments for genomic research [28] and is strongly advocated by the HUGO Committee on Ethics, Law and Society [29]. Yet, the acceptability of this approach is also attached with provisions for governance mechanisms that ensure transparency and accountability in how data are stored and shared with other researchers and institutions. Such mechanisms may include approval from an ethics review committee for specific projects, or a separate independent body comprised of members with relevant expertise to provide oversight for the release of data to external institutions and the distribution of benefits [7].

While the results of this study indicate that participants would consent to tumour profiling data being shared for the purposes of cancer research, this might not be limited to cancer research only as other types of biomedical research were not discussed in the interviews. However, the consent might not extend to non-medical related research, such as military research or forensic investigations. Concerns over the use of genetic data for these purposes has been raised in the literature [30, 31], and while they are most relevant to germline research rather than somatic tumour profiling, participants are unlikely to understand these differences well enough to assume that they appreciate the risks of sharing these data. In these circumstances, institutions must assume a guardianship role to ensure that the data entrusted to them is not misused or perceived as such.

Finally, the concept of benefit sharing is another principle that has emerged to justify the use of broad consent regiments for genomic research [28] and is also endorsed by HUGO [32] Committee on Ethics, Law and Society. This principle does not imply that participants should benefit directly, as it is important not to promote therapeutic misconception. Rather, the principle prioritises benefits to be shared with *communities*. In the context of this study, the principle implies that mechanisms should be in place for the expedient dissemination of published research results as new discoveries in cancer treatments emerge and the reclassification of variants becomes clinically significant.

## Conclusions

As the integration of NGS to inform patient care is continually evolving in oncology practice, this is experience is novel to clinicians, researchers and especially to patients. Therefore, developing a framework for obtaining consent from participants for this type of testing becomes challenging when recommendations specific to tumour profiling worldwide are only emerging and there are no best practice principles explicit to genomic testing in a Singaporean context.

This study has highlighted that there is limited public awareness around cancer causation and genetics as well as an understanding of what informed consent entails. As genomics advances, communication of these concepts will become increasingly complicated, yet highly relevant to ensure realistic expectations of the test outcomes. It has become evident that support is required when tumour profiling tests are offered, from either a clinic co-ordinator or genetic counsellor, so that information and test outcomes are explained, ultimately ensuring that informed consent can be obtained in precision oncology settings.

## Additional file

Additional file 1: Participant Interview Guide. (DOCX 15 kb)

## Abbreviations

ASCO: American Society of Clinical Oncology
DSRB: Domain Specific Research Board
GA4GH: Global Alliance for Genomics and Health
ICGC: International Cancer Genome Consortium
NCCN: National Comprehensive Cancer Network
NCCS: National Cancer Centre Singapore
NGS: Next generation sequencing
OECD: Organisation for Economic Co-Operation Development
PARP: ADP-ribose polymerase
TCGA: The Cancer Genome Atlas
WHO: World Health Organization
