# Supplementary material for: Ethical frameworks for obtaining informed consent in tumour profiling: an evidence-based case for Singapore
Source: Hum Genomics. 2017 Dec 8;11:31. doi: 10.1186/s40246-017-0127-1 (PMC5723075; doi:10.1186/s40246-017-0127-1)
Supplement: Additional file 1: — Participant Interview Guide. (DOCX 15 kb) [file 40246_2017_127_MOESM1_ESM.docx]

**Supplementary 1: Participant Interview Guide**

**Clinicians**

1. **Cancer Diagnosis and Genetics**
   1. Could you speak about your experience of how patients receive and understand their cancer diagnosis?
   2. Does anything come to mind when you hear the words "genetic or genomic"
   3. Do you discuss with your patients how genetic mutations may play a role in cancer development?
      1. Can you explain the difference between germline and somatic mutations?
2. **Tumour Profiling test**
   1. Can you tell me how you envision this test being incorporated into clinical practice?
      1. Who should deliver the information about the clinical and research components of the test?
      2. Do you feel that you know enough about somatic mutations in your patients' cancers to adequately explain this test?
      3. How will patients feel about the research component of the test? What sort of information would they need in the informed consent documents?
3. **Model of Informed Consent for Tumour Profiling**
   1. Which type of informed consent model would be best suited for the test?
   2. What concerns would you have on data security, governance structure and future research protocols that would be critical to patients and health care providers. How should they be discussed in the informed consent document?
4. **Storing and Sharing of Data**
   1. What are your thoughts on how this should be framed in the informed consent documents?
   2. From your experience, do you envision patients having concerns with storing of their tumour genetic data for an unidentified time frame? Sharing of their de-identified data both locally and globally?
   3. Can you discuss the role of patient empowerment in clinical and research decisions for sharing of molecular data?
5. **Possible germline findings from tumour profiling**
6. How would you manage germline incidental findings (i.e. BRCA)? How do your ideas about the type of informed consent, return of new findings and infrastructure requirements change with the incorporation of germline testing?
7. In the event of germline implications, how would your ability to provide patient support alter---are there any things that would need to be done differently between somatic vs. germline implications when returning findings?
8. What should be done with information gathered from germline testing if patients do not want to know the results?

**Breast cancer patients**

1. **Awareness around genetics, cancer and informed consent**
   1. Please tell me your thoughts on what are genes or genetics? How do you think cancer develops?
   2. Have you ever heard of genetic testing for cancer? What did you hear and where?
      1. When you think about cancer, genes or mutations are there certain words or ideas that come into your mind?
   3. What are your thoughts / beliefs on genetic testing?
      1. What happens if you were ill and had the option of getting a genetic test to understand how your disease developed? Would you want this disease specific genetic test to personalize your treatment? Why or why not?
   4. What is your understanding of informed consent?

**Research and informed consent**

1. Do you understand the differences between the clinical and research?
2. What will happen to your genetic data?
   1. Discuss about ways to keep data privacy?
   2. What are the possible risks?
   3. What are the possible benefits?
3. How do you feel about the idea of your genetic data being stored indefinitely? - *How do you feel about it being stored in Singapore? How do you feel about it being stored outside of Singapore and accessed by researchers in other countries?*
4. Would you like to find out about the results of the test?
5. What happens if you change your mind about sharing and storing of my genetic data?
6. Would you want to participate in research like this?
   1. Why or why not?
   2. What sort of additional information would you want to know
   3. If no, what kinds of information would make you change your mind about the research participation?
7. If researchers or this test discovered something serious about your heath, do you think they should let you know?
